# Round the Hospitals

**Published:** 1918-06-29

**Authors:** 


### ROUND THE HOSPITALS.

IT will be remembered that the St. John Ambulance Brigade Hospital at Etaples was last month wantonly bombed and afterwards bombarded by twenty German aeroplanes, which succeeded in dismantling the hospital and killing a number of its staff and occupants. The following members of its nursing staff have been awarded the Military Medal: Miss C. E. Todd, matron; Miss M. A. Chittock, assistant matron; Miss M. McGinnes, sister; Miss M. H. Ballance, sister; Miss J. Bemrose, sister; Miss C. Warner, sister. Miss Todd, who was trained at Guy's Hospital, is the matron of St. James' Infirmary, Balham, and Miss Chittock was previously assistant matron at Guy's Hospital. We offer our congratulations to all these ladies on the well-merited honour that has been conferred upon them. The medals will serve to remind the recipients of the awakening of the whole of the civilised world, by the outrage they suffered, to the devilish cruelty and murderous practices to which the German airmen have shown themselves to be willing to resort at the wish and call of the All-Highest and his officers. The scene in the House of Commons on the 25th instant, as reported in the *Times* of the 26th June, page 8, caused by the defeatists and miscalled pacifists, who ought to be in the service of the enemy, led to a proper rebuke by Commander Hamilton Benn, the member for Greenwich. His declaration, "I look upon the action of many members of the House who sit here below the gangway as that distinctly of traitors," expressed the view which is generally held throughout Great Britain and the Empire. Is the House of Commons so impotent, and is British law so faulty, that such proceedings may be suffered and can be allowed to continue in the present House

☛ Previous articles appeared on June 8 and 22, p. 255.

of Commons at the option and will of Germany and her traitorous backers? The constituencies concerned are at any rate British. Is it not time for the whole of their inhabitants of both sexes to rise, repudiate, and reject the traitors.

A LARGE number of awards of the Royal Red Cross has been made by the King to matrons and sisters. A Bar to the Royal Red Cross was awarded to Miss C. H. Keer, R.R.C., Matron-in-Chief of Q.A.I.M.N.S. (ret.); to Miss A. Nixon, R.R.C., matron of the Royal Herbert Hospital, Woolwich; and to Miss Yorke, R.R.C., matron of the Red Cross Hospital, Winchester. The Royal Red Cross, First Class, has been bestowed on thirty-seven matrons and sisters, while about 370 have been admitted Associates of the Order. We note a large number of Canadian, Australian, New Zealand, and South African recipients.

MISS LUCY JOLLEY, A.R.R.C., who has just been appointed Matron-in-Chief of the Air Service, was matron of the Royal Southern Hospital, Liverpool, from 1910 up to her resignation a few months ago. On the outbreak of war she was called up as a member of the Q.A.I.M.N.S.R., and left England almost immediately, first serving on an ambulance train and later at a casualty clearing station. She has lately resigned from the Reserve. At the recent College of Nursing Conference Miss Jolley's address on "Impressions of Nursing at the Front" created great interest, and showed her to have a wide grasp and knowledge, not only of her profession, but of nursing under many circumstances at the Front. Miss Jolley, on completing her training at Guy's Hospital, occupied the positions of ward sister, then of night superintendent, and then of instructress to the preliminary training-school. She also served on the matron's office staff. She has thus had a wide training in many branches of her profession. The appointment of Matron-in-Chief of the Air Service is a new one, and marks an important departure in its organisation, for this Service will now have its nursing arrangements under its own control. It is important, therefore, that the new Matron-in-Chief should have a wide experience and knowledge of her profession, and the brief account we have given of Miss Jolley's career demonstrates that she has had the good fortune to enjoy full opportunities for equipping herself adequately for the important post she is now called upon to occupy.

THE Procession of Homage to the King and Queen on Saturday to present an address on the occasion of their silver wedding will be formed of officers and women in uniform to the number of 3,000, who are whole-time workers in State departments under local authorities. The units represented include among others the Joint Women's V.A.D. Department, Women's Royal Naval Service, Women's Legion, Queen Mary's Army Auxiliary Corps, Women's Land Army, Women's National Land Service Corps, munition workers, and the Women's Police Service. A detachment from the Endell Street Hospital will walk in the procession, with Dr. Amy Sheppard as their representative. Their Majesties will receive the address of loyalty and homage at Buckingham Palace at 3 P.M., Miss F. H. Durham, Chief Inspector of the Ministry of Labour, having been chosen to read it. After replying to the address the King and Queen will receive the head of each corps and a representative woman from each section. Miss Eleanor Fortescue Brickdale has painted a symbolic picture of women war workers, with the address in coloured lettering beneath.

PRINCESS MARY has been stimulated by the course of First Aid and Home Nursing in which, as we mentioned a short time ago, she has obtained certificates, to advance a step further in the practice of the nurse's art. She has now begun a regular course of practical nursing work at the Children's Hospital, Great Ormond Street, where she attends twice a week, working in the Alexandra Ward. Here she learns, like other probationers, how to wash and dress the babies and tend the older little patients. It is an example of immense value at the present time, and should give a lead in quarters where reluctance is still sometimes shown by parents to allowing their girls to make that stride forward from theory to practice which tests character and incidentally puts forward the work of the world.

THE Society for the State Registration of Trained Nurses, a lengthy title which has long done duty for the absence of strength and authority—held what we believe to be its first public meeting at 11 Chandos Street, W., on the 20th instant. The financial statement showed receipts of £181, payments of £105, and a balance in hand of £76. After the adoption of the report with the financial statement the executive committee was elected *en bloc*, including recently appointed representatives of various bodies. Major Chapple, M.P., congratulated the Society on the progress made in the direction of State Registration. He declared that in these days everybody was a State Registrationist. He had been for a long time in communication with Sir Arthur Stanley on the subject of the proposed joint Bill. This Society was not opposed to the College as a College in any way. But the College must reciprocate. He resented the so-called Nation's Fund for Nurses, which had no real connection with the College. The Fund was to be for the benefit of nurses who had served in the war. The College of Nursing was mainly for the benefit of women who were not yet nurses at all [a reference to the future so incomplete as to lack substance and grit]. Finally he came to the point, expressing the hope that it would yet be possible in the near future to go to the Government with an agreed Bill, which there was no doubt would be readily passed by both Houses of Parliament as a war measure.

**ROUND THE HOSPITALS**—(*continued*).

The conference held at the Women's Institute on the 21st inst. to discuss the future of the Nursing Profession brought together several educational authorities interested in the reorganisation of the profession, and Mrs. Alderton, Principal of a Girls' High School, took the chair. She deplored the lack of prospects offered to girls at the outset of life by the nursing profession, and put forward the proposal that a resolution embodying suggested improvements in details of training likely to make the profession more attractive for educated women should be sent round to hospital governors. Miss Saunders, of the Horticultural College, exposed the need for greater attention to the study of dietetics in the training of the nurse. Much can be learned by the frank comments of educational people on the hospital training system. Matrons need their co-operation and cannot afford to disregard their criticism.

Mrs. Bedford Fenwick enforced the need for State Registration as the panacea for all ills. Miss Cowlin, Organising Secretary of the College of Nursing, agreed as to the need for legislation, but maintained that State Registration could not by itself make the nursing profession what it ought to be. "It must be guided by a united and expert body." She illustrated her argument by facts observed in the United States, where in many quarters the States' Bills were a dead letter. Unfortunately, at this point in the conference "the seven laymen," like King Charles' head, became mixed with the proceedings, and once they appear no sensible talk can make itself heard. It is a pity that no resolution was moved to give point to the discussion.

To obtain **The HOSPITAL** regularly **EVERY THURSDAY** a definite order **must** be placed with your Newsagent **at once.** All Newsagents, Booksellers, and Railway Bookstalls will gladly book your order, but copies cannot be stocked for sale to chance customers.

The total number of British Red Cross Auxiliary Hospitals with sick and wounded soldiers still under treatment amounted at the end of May to 1,172, the number of available beds being 64,448. This is a net increase of 1,269 beds during the month. No fewer than 65,985 women now belong to the Voluntary Aid Detachments registered with the War Office. A new regulation provides that members who have served three months' whole-time consecutive service in a hospital may enter for First Aid and Home Nursing examinations without attending the usual course of lectures. This is a sound concession. No sensible woman can fail to learn more in her first week in hospital than in the lectures prescribed for the help of amateurs, and all should be thoroughly well fitted in a month or two to master the text-books set for examination.

The action of the authorities of the University College Hospital in instituting what is practically an eight hours' day for their nurses is another notable indication that the hour for reform in nursing matters has sounded. Under the new arrangement nurses have 1 hour 40 minutes per day less duty than formerly, or a total of a little over 11 hours extra time off duty during the week. Sir Ernest Hatch, chairman and treasurer of the hospital, in advocating the shortened hours, declared that the public would not be losers by the extra cost entailed in making this alteration. We believe, on the contrary, that, regarded as employers, the public will soon have evidence that they, and those for whose benefit they subscribe, are gainers by it. In all directions managers of commercial affairs are discovering that shortened hours mean increased efficiency and a larger output. It is reasonable to conclude that philanthropic undertakings will react to the same extent under an improvement in the working hours of employees.

The report on the cost of a military patient in civil hospitals, prepared by the British Hospitals Association and lately reviewed in our columns, sums up strongly against the prevalent system of having a trained nurse as housekeeper. Stress is laid on the far greater importance of her catering than of her nursing qualifications, and the important point is emphasised that the trained sister, however competent she may become in the affairs of her department, tends to be but a passing occupant of her post. Few fully trained nurses are content to remain for very long so completely cut off from their art as the housekeeping sister must necessarily be. In fact these posts become more and more coveted as the passport to further promotion. Their value in preparing for matronships may conceivably be held to justify the appointment of sister-housekeepers. It is a recognised stepping-stone in the training-school. But if economy be the desideratum, then it cannot be denied that the promotion of successive sisters who must learn their art at the expense of the institution is not the way to compass it. The report alluded to above remarks: "We were glad to observe that this practice, formerly almost universal, is being superseded by a more common-sense one."

The American Navy Nurse Corps is very rigid in its requirements. Miss Lenah S. Higbee is the superintendent, and we learn from the *American Journal of Nursing* that she has intimated that candidates for admission to the Service must have "graduated" from a large general hospital, must be State registered, must be full citizens, and eligible for membership of the American Nurses' Association. In addition to this, they must pass "a professional and mental examination" under a board appointed for this purpose. For Army purposes both State registration and membership of the American Nurses' Association have been waived for the period of the war.

**ROUND THE HOSPITALS—(*continued*).**

IN forming local Centres of the College one of the chief difficulties is to ascertain what College members are residing within the area and to get into touch with them. The London headquarters cannot possibly tell what registered members may happen to be working in a given locality, and nurses are liable to be overlooked. The Yorkshire Centre of the College at Leeds appeals to all College members in that area to communicate either with Miss Spann, of the Township Infirmary, Leeds, who is the secretary for all Poor-Law infirmaries and fever hospitals, or with Miss Lindall, Hospital for Women and Children, Leeds, who is the secretary for all hospitals and nursing associations in the county. Nurses should give their names in full, their present address and position, their permanent address, the name of their training-school, with date of certification, and date of enrolment on the College Register. The local subscription has been fixed at 2s. 6d., which should be enclosed with the application. It is proposed to give post-graduate lectures in the autumn in the clinical theatre of the Leeds General Infirmary, and to these annual subscribers to the College will be admitted free.

---

THE Commissioners appointed to inquire into the administration of the Beckett Hospital, Barnsley, and into the causes of unrest (if any) among the nursing staff during the eighteen months prior to December 1917, have now presented their report. The public showed little interest in the proceedings, and not one of the late staff appeared to give evidence of any unfair treatment. The Commissioners reported that there was no unrest among the nursing staff during that period and no cause for unrest; that when the present matron was appointed in September 1916 "there was some evidence that the discipline of the nursing staff had become relaxed, but discipline was restored without friction and without delay." They found at the present time the staff management satisfactory. In conclusion the Commissioners expressed the opinion that "a room should be provided for the nurses, in addition to the present recreation-room, where they could enjoy the quiet and silence necessary for study, serious reading, and other intellectual pursuits." This finding is eminently satisfactory both for subscribers and for the matron. The circumstances connected with holding this inquiry illustrate the importance of giving in the yearly report such a summary of nursing matters as is afforded by some of the major training-schools—viz. the number of new probationers engaged, the number who left the institution without receiving a certificate, with the causes why they did not complete their term, and the work taken up by the newly certificated nurses. It is due to subscribers to know these facts, and good for the institution that they should be supplied.

PLYMOUTH has been making fête from the hospital point of view, two charming receptions having taken place within a day of each other on June 13 and 15. The first event was the garden-party at the 4th Southern General Hospital, Home Lodge, given by Miss Tait McKay and her staff to the officer patients of Hyde Park Section, and others interested in the hospital. A tennis tournament was the event of the afternoon, and the Band of the Royal Garrison Artillery enlivened the proceedings. A distinguished company gathered in the grounds, and the matron and her staff received many compliments on the success of their reception. On the 15th inst. Plymouth witnessed a march of the American soldiers, the medical and general staff of the United States Military Hospital, situated in the Laira and Efford hutments on the outskirts of Plymouth, being invited by Major-General Sir Victor A. Couper, K.C.B., to a garden party at Government House. The principal officers and matrons of the Military and Territorial Hospitals, Miss Monck-Mason, Miss Tait McKay, and Miss Margaret Priestman, were invited, with the senior naval and military officers in port or garrison, to take part in the reception, which was attended by the Naval Commander-in-Chief, Sir A. E. Bethell, and Lady Bethell, the Mayor of Plymouth, and other notable persons. About 140 United States' nurses were present, with their matron, Miss Mack, and their appearance in smart, business-like, navy blue costumes and hats was highly approved. They were accompanied by their dietician and secretary, both civilians.

---

AN important judgment was given on June 18 by Mr. Justice Roche in an action brought by Mrs. Margaret Davies, of the Dorrien Nursing Home, North Finchley, against Miss Ada Ward, claiming damages for having enticed Gladys Clappen to quit her employment as a professional nurse. Miss Clappen, a qualified surgical nurse, was under contract to remain at the nursing home for the duration of the war, and received a salary of £28 a year, with board and lodging. She was sent out on two occasions for considerable periods to nurse at the defendant's house at a rate of £2 2s. for the first week and £1 10s. after, and eventually decided to leave the nursing home and accept a lighter post with Miss Ward. The judge decided that a breach of the law had been committed, inasmuch as the nurse was induced by the defendant to break her contract, and was offered material and substantial inducement to do so. He awarded Mrs. Davies £20 damages. Superintendents suffer a good deal, generally without resistance, under the "enticement" grievance, and it may be a surprise to some of them to learn that the law is on their side. Apart from taking legal proceedings, the designs of the public on good employees can best be countered by providing better salaries, shorter hours, and more agreeable conditions of service.

☛ For Nursing Affairs in Ireland: Sir Arthur Stanley's Visit—Who Has Been "Done"? see p. 284; Control of V.A.D. Hospitals, p. 290.

